# Effects of Mobile Text Advertising on Consumer Purchase Intention: A Moderated Mediation Analysis

**DOI:** 10.3389/fpsyg.2017.01022

**Published:** 2017-06-23

**Authors:** Lin Hongyan, Chen Zhankui

**Affiliations:** School of Economics and Management, Xiamen University of TechnologyXiamen, China

**Keywords:** mobile text advertising, infotainment, irritation, regulatory focus, attitudes, intention to purchase

## Abstract

Mobile shopping is increasing in prevalence and has become a necessary part of many people's daily lives. However, one main channel for mobile shopping, mobile shopping applications (apps), has not been thoroughly investigated. This study focused on mobile text advertising delivered from mobile shopping apps using the intention to purchase as the dependent variable for testing its marketing effect. In the context of a promotion focus vs. a prevention focus, we used Higgins' regulatory focus theory combined with Ajzen's TPB and Herzog's U&G to analyze the mechanism by which consumers formulate an intention to purchase in a mobile advertising context. This empirical study surveyed 320 consumers who had made a purchase using a mobile shopping app in the previous month. The results showed that infotainment, irritation, and subjective norms were significantly associated with attitudes; in turn, attitudes mediated the impact of these three factors on the intention to purchase. Moreover, a high promotion focus not only strengthened the positive effect of infotainment on attitudes but also intensified the mediation effect of attitudes between infotainment and the intention to purchase. A high prevention focus also consolidated the negative effect of irritation on attitudes as well as reinforced the mediation effect of attitudes between irritation and the intention to purchase. Furthermore, attitudes, subjective norms, and perceived behavioral control collectively impacted the intention to purchase. These findings shed light on ways to customize goods information in mobile advertising and have strong theoretical and practical implications.

## Introduction

With the rapid development of the mobile internet, consumers use smartphones not only for communication but also for other activities, such as surfing the web and participating in mobile business. Mobile shopping has become a natural extension of traditional online shopping, thereby achieving multi-scene synchronous shopping (Ngai and Gunasekaran, 2007). According to the latest statistics from the China Internet Network Information Center(CNNIC, 2017), Chinese cell phone cybercitizens now number 695 million, which is 95.1 percent of the total number of people in China who use the internet. Moreover, 441 million users are mobile shoppers. Online shopping platforms, such as Amazon, Taobao, and JD, have exerted considerable effort toward developing mobile business, including developing mobile shopping applications (apps) (Yang et al., 2015). In general, downloading these apps can only be done with the permission of users. Thus, the mobile apps in a user's smartphone have generally been downloaded voluntarily. Such apps are widely used (Lee and Santanam, 2014). For example, Taobao is an open market similar to Amazon. It was developed by the largest Chinese retail trading platform, the Alibaba Group. The Taobao app was launched in 2011. Chinese mobile apps have gradually become commonplace in the past few years. Furthermore, a number of apps facilitate the delivery of advertising content (Grewal et al., 2016). A statistical analysis showed that the United States had more than $19 billion in investments in the mobile advertising market in 2015, a figure expected to exceed $65 billion by 2019 (eMarketer, 2015). The growth of the world market is similar to that of the United States. eMarketer (2015) predicted that $100 billion will be invested in mobile advertising in the world market by 2016. Yet, to our knowledge, there are no previous studies on the effectiveness of mobile text advertising from apps.

Smartphones and other mobile devices have achieved a high degree of personalization and have become vital communication tools; most users constantly keep them nearby, even when they sleep (Bacile et al., 2014). Such devices grant consumers ubiquitous access to digital information at any time and any place; thus, these devices also allow marketers a great opportunity to reach consumers directly and constantly and to analyze their needs (Grewal et al., 2016). Hence, these devices make personalized advertising services possible. But, the consumer's attention is limited. Companies that sent a large number of advertisements to users found that this was not a good marketing method (Stewart and Paul, 2002). The issue of how to optimize the sending of suitable mobile advertising to targeted users is thought-provoking.

Most research on mobile advertising used either the theory of reasoned action or the theory of acceptance model to examine the influence of a technique's characteristics, (e.g., its perceived usefulness and perceived ease of use), information characteristics, and perceived value on consumers' attitudes, adoption, or intention to use (Tsang et al., 2004; Kim et al., 2007; Liu et al., 2012; Muk and Chung, 2015). However, a few empirical studies have developed their mobile advertising research from the perspective of other individual characteristics, such as regulatory focus. Recent sociological studies indicate that the regulatory focus level of individuals considerably affects their behavior, a finding which has been partially validated for environmental advertising (Bhatnagar and McKay-Nesbitt, 2016). But, studies about the impact of commercial mobile advertising on the intention to purchase have seldom been conducted (Bart et al., 2014).

**Figure 1:**
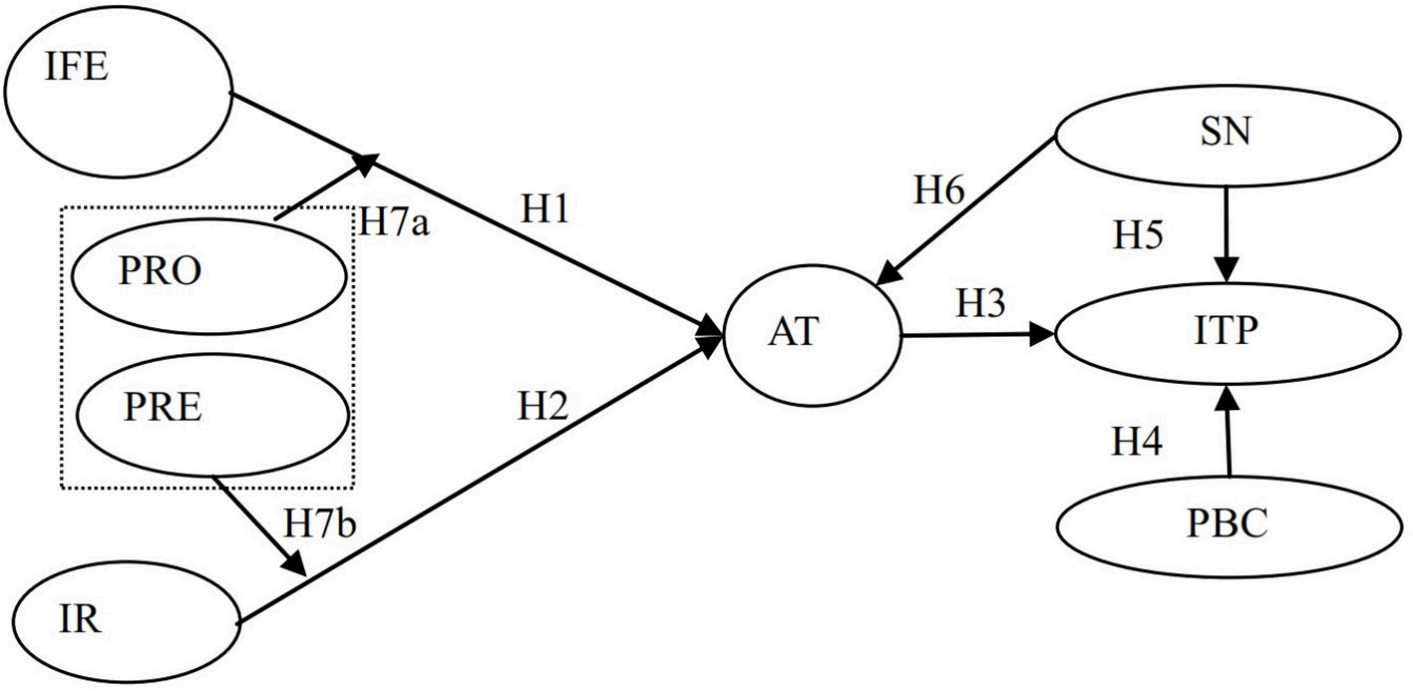
Research Model. IF = infotainment, IR = irritation, PR = promotion focus, PRE = prevention focus, AT = attitudes, SN = subjective norms, PBC = perceived behavior control, ITP = intention to purchase the advertised product.

This research attempted to tackle this deficiency by applying regulatory focus theory (RFT), theory of planned behavior (TPB), and uses and gratifications theory (U&G) to investigate the purchase intention of consumers after they had received commercial mobile advertising. The subjects were people who had recently purchased products using a mobile shopping app. The research model is shown in Figure 1. Specifically, (1) in the antecedents of attitudes section, as illustrated on the left side of the diagram, this study investigated whether infotainment and irritation would be the key factors affecting consumer attitudes toward mobile text advertising. (2) The outcomes section portrayed on the right side investigated whether the TPB applies to a mobile advertising environment, that is to say, whether consumer attitudes toward mobile text advertising, subjective norms, and perceived behavioral control influence a consumer's intention to purchase mobile advertised goods. (3) At the same time, based on the RFT, as also indicated on the left side of the model diagram in Figure 1, this study investigated whether a high promotion focus would not only strengthen the positive effect of infotainment on attitudes toward mobile text advertising but would also intensify the mediation effect of attitudes between infotainment and the intention to purchase. Would a high prevention focus also consolidate the negative effect of irritation on attitudes toward mobile text advertising as well as reinforcing the mediation effect of attitudes between irritation and intention to purchase? In addition, in the empirical section, the research model was examined by structural equation modeling (Anderson and Gerbing, 1988) and a bootstrapping method (Edwards and Lambert, 2007).

Different types of mobile advertising, such as video, multimedia, text advertisements, mobile display advertising, and short message service (SMS), are available. In general, the communication methods used in mobile advertising can be categorized into push and pull types (Park et al., 2008). (1) Push-based advertising is frequently delivered as an SMS. If a user is interested in the advertisement, s/he can click a link in the SMS to browse for additional information. (2) Pull-based advertising, which can be browser-based or within-app advertising, guides users to a mobile website and then provides interactive choices to understand their habits and preferences (Molitor et al., 2012; Zubcsek et al., 2016). The present study focused on push text advertising from a mobile shopping app for two reasons. First, such advertising is convenient for consumers to read and save, but, browsing a website and selecting goods generally take considerable time. However, push advertising is sent by companies and can be saved. Users can read these advertisements at any time. Second, permission-based advertising information can reduce consumer irritation (McCoy et al., 2008). Mobile push advertising is generally sent with the users' permission. Thus, push advertising is beneficial for forming a positive attitude and intention to purchase. Therefore, this study is consistent with actual marketing situations and can satisfy the practical needs of companies.

The contributions of this research are primarily in two areas. First, this study employed RFT, TPB, and U&G to explore the formation of consumer purchase intention in mobile shopping apps. Secondly, it tested for moderated effects and moderated mediation effects in the mobile text advertising context.

## Literature review and hypotheses

### Uses and gratifications theory (U&G)

U&G theory examines the psychological and behavioral utility produced by using mass communication and analyzes the use of motivating and need-satisfying information by audiences. Previous studies concluded that entertainment, informativeness, and irritation are vital dimensions of U&G (Ha et al., 2014). This theory has been widely used in consumer motivation research on traditional media and has made a substantial contribution to the field (Eighmey and McCord, 1998). Moreover, Ducoffe (1996) and Tsang et al. (2004) assumed that entertainment, informativeness, and irritation were related to consumer attitudes when browsing website and mobile advertisements. In this study, we investigated the impacts of informativeness, entertainment, and irritation on consumer attitudes after receiving mobile advertising from an app. This application to mobile advertising is a reasonable extension of U&G theory.

Entertainment and informativeness are interpenetrations in the new technologies (Wang and Sun, 2010). Hence, they can be integrated into one concept: infotainment (Okazaki, 2004). Studies show that whether the internet or mobile networks are involved, infotainment has a positive impact on attitudes, but irritation has a negative one (Okazaki, 2004; Shankar and Balasubramanian, 2009). Recent research also suggested that, as the intelligence of mobile functions increases, users will be able to increase their control of advertising information; consequently, the negative influence of irritation on consumer attitudes will also decrease (Watson et al., 2013). However, the way that these effects change has seldom been discussed. In this study, a promotion focus was investigated as a possible moderator of the positive effect of infotainment on attitudes toward mobile text advertising, whereas a prevention focus was investigated as possibly moderating the negative effect of irritation on attitudes toward mobile text advertising.

#### Infotainment

Information is a valuable incentive in mobile marketing because recipients of information tend to respond positively to advertising (Aitken et al., 2008). Therefore, informativeness, which primarily refers to the degree of information richness and the usefulness of a provided advertising media, is part of the infotainment construct (Ducoffe, 1996). Consumers expect information content that fits their interests (Robins, 2003) as well as information that is related to their own preferences and interests (Milne and Gordon, 1993) when they connect with mobile services. Some researchers have maintained that receiving suitable information from advertisers does not anger consumers (Varshney, 2003). At the same time, other researchers have shown that if advertising providers provide pleasant, relevant, and informative advertisements, the consumers' intention to purchase may increase (Scharl et al., 2005). Entertainment can deeply move customers and further familiarize them with the available services or goods (Liu et al., 2012). Thus, entertainment constitutes the other aspect of the infotainment concept.

Early research revealed that interesting and pleasurable advertising can actively influence attitude (Shimp, 1981). Moreover, a feeling of enjoyment was found to be central to a consumer's attitudes (Shavitt et al., 1998). Zhang and Mao (2008) held that perceived informativeness and perceived entertainment were components of perceived utility, and Rohm et al. (2012) explored the issue of whether perceived utility was a determinant of attitude toward mobile marketing. Furthermore, recent research showed that advertisements with more information than usual and a high level of entertainment can be readily accepted (Lin et al., 2014, 2016).

In this study, we investigated the attitudes toward mobile text advertising (branded communication). This topic is worth exploring because mobile advertising is a new and effective form of media advertisement. According to the latest forecast by eMarketer (2017), most of the Chinese advertising expenditures in the future will be invested in mobile advertising. Because a mobile device is a private good, such advertising can easily be delivered to targeted customers. At the same time, because it can conveniently be accessed and saved, mobile text advertising via apps has become a common marketing tool for a number of companies. Thus, discussing customers' attitudes toward mobile text advertising is valuable.

Furthermore, users seem to feel that voluntarily downloaded apps are useful and valuable (Kang, 2014), and their attitudes toward them may be positive, even without any mobile text advertising. In addition, users' attitudes toward branded products are heavily influenced by their attitudes toward mobile advertising (Drossos et al., 2013). According to U&G theory, if users are satisfied with one type of mobile advertising, they will be likely to accept the included products brand information and buy the product. Therefore, attitudes toward mobile text advertising are the determinant of the intention to purchase. Focusing on the attitudes toward mobile text advertising is, therefore, worthwhile and suitable.

Based on the above, Hypothesis 1 was:

H1: Upon receiving a text advertisement from a previously voluntarily downloaded shopping app, consumers' attitudes toward mobile text advertising will be positively related to their perception of the infotainment level of this text advertisement.

#### Irritation

Irritation in the context of advertising can be described as the way that such advertising annoys consumers (Liu et al., 2012). The primary cause of criticisms of advertising is that it is an irritation (Bauer and Greyser, 1968).

When mobile advertising first began, the negative impact of irritation on consumer attitudes was considered to be great. “When advertising employs techniques that annoy, offend, insult, or are overly manipulative, consumers are likely to perceive it as an unwanted and irritating influence” (Ducoffe, 1995). Subsequently, Siau and Shen (2003) claimed that common people were unfamiliar with the concept of mobile commerce and were skeptical about the feasibility and security of mobile advertising. Moreover, when consumers received an excessive number of advertisements from a provider, it disturbed them (Bruner and Kumar, 2007). More recently, with the improvement of the smartphone, others argued that, although the irritation of SMS negatively affects attitudes in a mobile context (Liu et al., 2012), the extent is less than before (McCoy et al., 2008). Thus, the following hypothesis was formulated:

H2: Upon receiving a text advertisement from a previously voluntarily downloaded shopping app, consumers' attitudes toward mobile text advertising will be negatively related to their perception of the irritation level of this text message.

### Theory of planned behavior (TPB)

Ajzen (1985) proposed a general model, the TPB, to explain the unique behavior of individuals. It reflects an individual's beliefs, attitudes, and intentions. The TPB is an extension of the theory of reasoned action (Ajzen and Fishbein, 1975, 1980). In the expectations–value model (Fishbein and Ajzen, 1974), attitudes are considered to be the consequence of individual beliefs. Subjective norms refer to the extent that the perception of opinions from his/her reference groups, such as friends and colleagues, impact an individual's behavior (Schofield, 1974). Perceived behavioral control refers to an individual's subjective appraisal of their own ability and their opportunities to carry out a specific behavior (Ajzen and Madden, 1986). Intention is another vital indicator of an individual's behavior (Ajzen, 1991). The TPB holds that an individual's behavior is impacted by her/his beliefs, attitudes, subjective norms, and other uncontrollable factors (Crespo and Bosque, 2008). In this study, these three variables were used to investigate the consumer's intention to purchase the advertised product. These variables have been used in several previous studies to explore online consumer behavior intention (Luarn and Lin, 2005; Kim et al., 2011; Lin and Chen, 2015).

An individual's attitude tends to be a determinant of his/her behavioral intention (Ajzen and Fishbein, 1975). Many studies have showed a positive relationship between the attitude toward an advertisement and an intention to purchase (Drossos et al., 2013). Subjective norms were found to play an important role in predicting people's intention to consume fast food (Bagozzi et al., 2000) and have been perceived as positively shaping young Chinese people's intention to use mobile services (Zhang and Mao, 2008). Following the TPB, this study assumed that attitudes, subjective norms, and perceived behavioral control affect a consumer's purchase intention. Moreover, attitude is typically influenced by the opinions of others (Kim et al., 2013). Thus, the following hypotheses were investigated:

H3: Upon receiving a text advertisement from a previously voluntarily downloaded shopping app, consumer attitudes toward mobile text advertising will have a positive influence on their intention to purchase.
H4: Upon receiving a text advertisement from a previously voluntarily downloaded shopping app, consumer's intention to purchase will be positively impacted by their perception of their degree of behavioral control.
H5: Upon receiving a text advertisement from a previously voluntarily downloaded shopping app, consumer's subjective norms will impact their intention to purchase.
H6: Upon receiving a text advertisement from a previously voluntarily downloaded shopping app, a consumer's subjective norms will predict their attitudes toward mobile text advertising.

### Regulatory focus theory (RFT)

RFT is a goal pursuit theory. It was articulated by Higgins et al. (1997) to address people's perceptions during their decision-making process. Higgins (2000) believed that the consumer decision process stems from a variety of motives. Motivation can be divided into two types: one is to achieve a desired goal, and the other is to avoid an appearance of negative results (Lockwood et al., 2002; Das, 2016). RFT proposes that regulatory focus is a special propensity of individuals when they self-regulate to achieve their goals (Higgins et al., 1997; Brockner et al., 2004). RFT is a chronic manifestation and includes promotion focus and prevention focus, two independent orientations. Promotion focus refers to people's motivation to seek pleasure and is related to an ideal target, that is, that they can obtain a desired outcome. During self-regulation, a radical strategy is required to maximize the probability of obtaining a positive outcome, such as improving the benefit (Crowe and Higgins, 1997; Freitas, 2002). Meanwhile, prevention focus refers to people's motivation to avoid pain and is related to a “should be” target, that is, that they should experience no loss or punishment. A prevention focus usually generates a cautious strategy to minimize the occurrence of a negative outcome, such as reducing a loss (Crowe and Higgins, 1997; Kirmani and Zhu, 2007; Lee et al., 2014). Although a person's regulatory focus is a long-term personality trait, it can be triggered to impact a temporary motivation in a specific situation (Wilson and Ross, 2000; Lockwood et al., 2002).

RFT has been widely used in the field of consumer behavior (Avnet and Higgins, 2006; Wang and Sun, 2010; Ouyang et al., 2015). Scholars have found that promotion and prevention focuses significantly influence consumer decision making (Lee and Santanam, 2014; Das, 2016). According to RFT, a prevention focus causes consumers to perceive a loss from utilizing bundled sales to be higher than it really is (Lin and Huang, 2011), whereas a promotion focus encourages consumers to be more mindful of future results (Joireman et al., 2012). Furthermore, individuals' promotion and prevention focuses have different effects on their intention to purchase, on their participation in online product reviews, and on their willingness to spread a positive word of mouth (Das, 2016). Most of these studies primarily involved Western consumers and are more directed toward traditional and internet marketing environments. Relatively little research exists on Asian consumers in the mobile marketing environment (Zhang and Mao, 2008; Lin and Chen, 2015). This study focused on the different motivations of Chinese consumers in response to mobile text advertising and explored the manner in which a promotion focus or a prevention focus can affect purchasing decisions.

#### Moderation effect

According to RFT, when the type of regulatory focus matches the implemented strategy, individuals evaluate that strategy highly and have positive attitudes (Wang and Lee, 2006). Therefore, some researchers proposed that companies should emphasize different aspects when facing different consumers. Specifically, acquiring greater potential revenue will attract promotion focus consumers, whereas avoiding potential losses will draw prevention focus consumers (Higgins, 2000; Wang and Lee, 2006; Park and Morton, 2015). Moreover, other scholars argued that a high level of promotion focus will result in the consumers' high evaluation of hedonic goods (Hassenzahl et al., 2008). Thus, we supposed that high promotion focus individuals will be inclined to take positive measures to add to their enjoyment; thus, the influence of infotainment on attitudes will be intensified. However, people with a high prevention focus will tend to take protective actions to avoid possible loss, thus the negative impact of irritation on their attitudes will be strengthened. The hypotheses are as follows:

H7a: Upon receiving a text advertisement from a previously voluntarily downloaded shopping app, a high promotion focus will strengthen the positive relationship between infotainment and attitudes toward mobile text advertising.
H7b: Upon receiving a text advertisement from a previously voluntarily downloaded shopping app, a high prevention focus will strengthen the negative relationship between irritation and attitudes toward mobile text advertising.

#### Moderated mediation effect

Consequently, promotion focus consumers will be inclined toward using promotion strategies to increase pleasure and attain their goals, whereas prevention focus consumers will tend to use protection strategies to avoid losses. In particular, the infotainment of mobile text advertising can be expected to allow the promotion focus consumers to form more positive attitudes and a greater intention to purchase, whereas irritation stemming from mobile text advertising will generate more negative attitudes and reduce the intention to purchase in prevention focused individuals. Thus, we assumed:

H8a: Upon receiving a text advertisement from a previously voluntarily downloaded shopping app, having a promotion focus will moderate the mediation effect of attitudes toward mobile text advertising between infotainment and the intention to purchase, such that, when the promotion focus is high, the mediation effect of attitude will be strong.
H8b: Upon receiving a text advertisement from a previously voluntarily downloaded shopping app, having a prevention focus will moderate the mediation effect of attitudes toward mobile text advertising between irritation and the intention to purchase, such that, when the prevention focus is high, the mediation effect of attitude will be strong.

## Research design

One common form of current commercial activity—mobile shopping apps—was selected as the source of mobile advertising information. A survey that was aimed to measure their response was given to a group of consumers after they received a text advertisement. An electronic questionnaire was used for data collection. Each participant was chosen randomly if he or she had previously had a shopping experience with a famous Chinese mobile shopping app, Taobao, and had purchased products from the same app at least once in the previous month.

### Data collection

The investigated app was the Taobao app. Its usage percentage in China was 24.1% by the end of 2016 (CNNIC, 2017). By the latest statistics from China Internet Network Information Center (CNNIC, 2017), the structure of total Chinese cybercitizens is as follows: the male to female ratio is 52:48; 30.3% are aged 20–29, 23.2% are aged 30–39; 11.5% hold a bachelor's degree; 25% are students, and 64.3% are currently employed.

According to investigational results from one Chinese mobile data business service company, Questmobile, the Taobao app had up to 406 million subscribers by the first quarter of 2017 (QuestMobile, 2017). The demographic profile of its users in China, as of May 2016 was as follows: 49% male, 51% female and 72% of the users were aged 18–34. We have no reason to doubt that this is representative of Chinese shopping apps.

The number of people who responded to the questionnaire was 431, but 81 did not meet the two requirements after answering the first two questions, so they were not permitted to continue with the survey. Thus, a total of 350 respondents finished our survey with 320 valid questionnaires (a response rate of 74%). 171 (53%) were female, and 149 (47%) were male. Most of the participants were young; 240 (75%) respondents were aged 22 to 35 years. In terms of educational attainment, 234 (73%) of the respondents had a bachelor's degree. Moreover, 243 (76%) respondents were employed. The characteristics of these investigated data are broadly consistent with the total population of Taobao app users, so we believe that our data is a representative sample.

### Measurement

All of the measurement items in this study were adopted from previous research. The promotion and prevention focus scales are from Higgins et al. (2001) as translated by Ouyang et al. (2015) into a Chinese context to give them better credibility and reliability. This study directly used their scales to appropriately assess Chinese consumers. We translated the remaining scales from English to Chinese and implemented a back-translation to ensure the consistency and clarity of the questions' expression (Brislin, 1980). To examine the content validities, the translated questionnaire was assessed by two bilingual faculty members. A pretest was conducted to simplify the questions and to ensure that they precisely measured the concepts. Based on the feedback, confusing items were revised. The Cronbach's alpha for all the items was 0.89, which is considerably higher than 0.70, which was the level suggested by Hollis et al. (1978) as acceptable. The main variable measurement items are listed in Table 1.

**Table 1:** Main variable measurement items, reliability and factor loadings.

**Construct**	**Items**	**Standard factor loading**	**C.R**.	**AVE**	**α**	**Source**
**IFE**	**Infotainment**		**0.92**	**0.75**	**0.89**	Ducoffe, 1996
IFE1	The mobile text advertising was entertaining.	0.88				
IFE2	The mobile text advertising was enjoyable.	0.85				
IFE3	The mobile text advertising was pleasing.	0.87				
IFE4	The mobile text advertising was a good source of up-to-date product information.	0.85				
**IR**	**Irritation**		**0.95**	**0.87**	**0.93**	Tsang et al., 2004
IR1	Mobile text advertising is everywhere.	0.91				
IR2	The mobile text advertising was annoying.	0.94				
IR3	The mobile text advertising was irritating.	0.95				
**PRO**	**Promotion focus**		**0.85**	**0.65**	**0.73**	Higgins et al., 2001; Ouyang et al., 2015
PRO1	I feel I have often made progress toward being successful in my life.	0.81				
PRO2	I often try to reach the things in life in which I believe.	0.81				
PRO3	How often have you accomplished things that got you “psyched” to work even harder?	0.80				
**PRE**	**Prevention focus**		**0.95**	**0.87**	**0.92**	
PRE1	Growing up, did you ever “cross the line” by doing things your parents would not tolerate?	0.94				
PRE2	Did you get on your parents' nerves often when you were growing up?	0.93				
PRE3	Growing up, did you ever act in ways that your parents thought were objectionable?	0.93				
**AT**	**Attitudes toward mobile text advertising**		**0.91**	**0.78**	**0.86**	Alwitt and Prabhaker, 1994
AT1	Mobile text advertising helps raise our standard of living.	0.91				
AT2	Mobile text advertising helps me find products that match my personality and interests.	0.88				
AT3	Mobile text advertising helps me buy the best brand for a given price.	0.87				
**SN**	**Subjective norms**		**0.93**	**0.82**	**0.9**	Taylor and Todd, 1995
SN1	People who are important to me would think that I should use mobile online trading.	0.92				
SN2	People who influence me would think that I should use mobile online trading.	0.92				
SN3	People whose opinions are valuable to me would prefer that I use mobile online trading.	0.89				
**PBC**	**Perceived behavioral control**		**0.92**	**0.79**	**0.87**	Taylor and Todd, 1995
PBC1	I will be able to use mobile online trading well when trading.	0.89				
PBC2	Using mobile online trading is entirely within my control.	0.88				
PBC3	I have the resources, knowledge, and ability to use mobile online trading.	0.90				
**ITP**	**Intention to purchase**		**0.92**	**0.79**	**0.87**	Hwang et al., 2011; Bart et al., 2014
ITP1	I will be likely to purchase the advertised product.	0.90				
ITP2	I will purchase if it is necessary.	0.89				
ITP3	I will browse the online store to get what I want to buy.	0.88				

*N = 320; C.R. = composite reliability; AVE = average variance extracted; α = Cranach's alpha*.

Related research previously showed that demographic variables (gender, age, education level, and job) are among the important factors influencing attitudes (Wolin and Korgaonkar, 2003; Habuchi et al., 2005). Thus, these factors were used as control variables.

All the items were measured using a five-point Likert scale. The measurement for regulatory focus was from never (1) to always (5), whereas the others were from strongly disagree (1) to strongly agree (5).

## Data analysis and results

### Data analysis

#### Reliability and validity

All of the factor loadings were highly significant (*p* < 0.01) to their respective latent constructs; the composite reliabilities of all of the constructs were > 0.80; and all of the average variance extracted estimates were > 0.60 (Table 1; Fornell and Larcker, 1981). Therefore, the measurements had adequate convergent validity and composite reliability (Bagozzi and Yi, 1988).

#### Confirmatory factor analysis (CFA)

**Figure 2:**
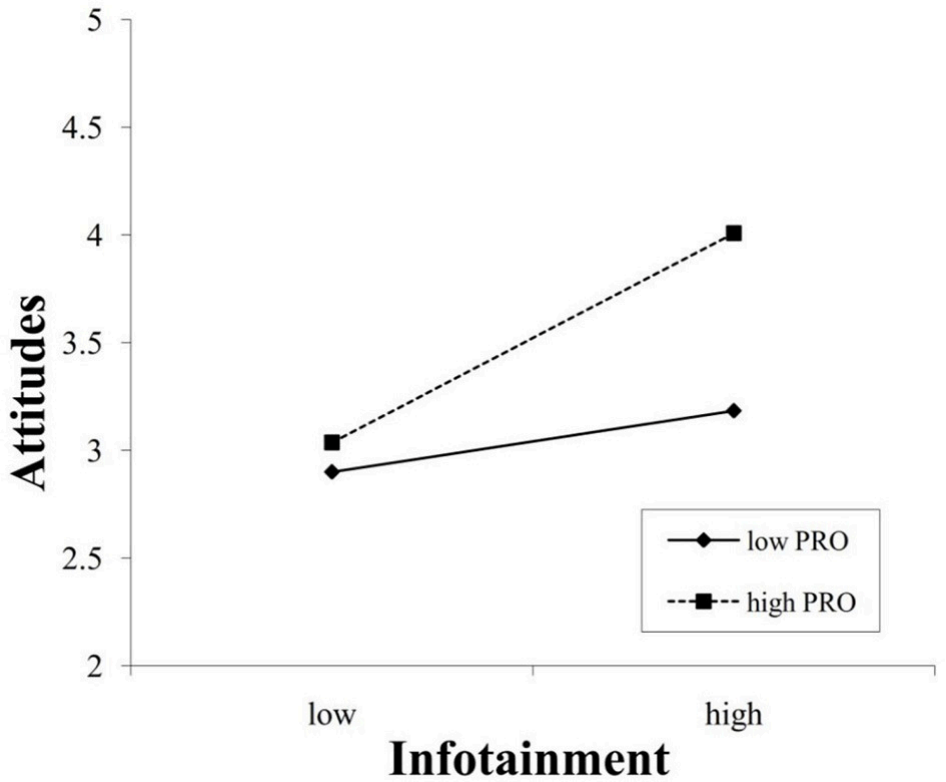
Interaction between infotainment and a promotion focus in predicting attitudes.

CFAs were implemented to examine the discriminant validity of the key variables. First, an eight-factor CFA model (see Table 2) was tested. This model included infotainment, irritation, promotion focus, prevention focus, attitudes, subjective norms, perceived behavioral control, and intention to purchase. The measurement model fitted the data (χ^2^ = 382.33, df = 247, TLI = 0.97, CFI = 0.94, RMSEA = 0.04), confirming the unidimensionality of the measures (Anderson and Gerbing, 1988). Subsequently, the eight-factor model was compared with alternative models that sequentially combined infotainment with each of the 7 other factors (Figure 2). A model comparison revealed that the eight-factor model fit the data considerably better than the alternative model. The means, standard deviations (SDs), and correlations are shown in Table 3.

**Table 2:** Results of the CFA for the measures of the studied variables.

**Model**	**χ^2^**	**Df**	**Δ χ^2^**	**TLI**	**CFI**	**RMSEA**
**Eight-factor model**	382.33	247	−	0.97	0.94	0.04
**Seven-factor model-1**:	1174.35	254	792.02^**^	0.78	0.81	0.11
Infotainment and Irritation combined						
**Seven-factor model-2**:	577.34	254	195.01^**^	0.92	0.93	0.06
Infotainment and Promotion Focus combined						
**Seven-factor model-3**:	1128.93	254	746.6^**^	0.79	0.82	0.10
Infotainment and Prevention Focus combined						
**Seven-factor model-4**:	944.31	254	561.98^**^	0.83	0.86	0.09
Infotainment and Subjective Norms combined						
**Seven-factor model-5**:	856.73	254	474.4^**^	0.85	0.88	0.09
Infotainment and Perceived Behavioral Control combined						
**Seven-factor model-6**:	826.95	254	444.62^**^	0.86	0.88	0.08
Infotainment and Attitudes toward Mobile Text Advertising combined						
**Seven-factor model-7**:	876.27	254	493.94^**^	0.85	0.87	0.09
Infotainment and Intention to Purchase combined						
**One factor model**:	3251.18	275	2868.85^**^	0.27	0.33	0.19

N = 320;

** *p < 0.01 (2-tailed)*.

**Table 3:** The means, SDs, and correlations of the main variables.

	**Mean**	***SD***	**(1)**	**(2)**	**(3)**	**(4)**	**(5)**	**(6)**	**(7)**	**(8)**
(1) Infotainment	3.48	0.85	(0.86)							
(2) Irritation	2.94	1.19	−0.13^*^	(0.93)						
(3) Promotion Focus	3.76	0.66	0.26^**^	−0.07	(0.81)					
(4) Prevention Focus	2.02	1.04	−0.17^**^	0.02	−0.18^**^	(0.93)				
(5) Attitudes toward Mobile Text Advertising	3.33	0.91	0.43^**^	−0.31^**^	0.33^**^	−0.35^**^	(0.88)			
(6) Subjective Norms	3.12	0.97	0.28^**^	−0.19^**^	0.22^**^	−0.17^**^	0.47^**^	(0.91)		
(7) Perceived Behavioral Control	3.45	0.97	0.20^**^	−0.06	0.15^**^	−0.13^*^	0.35^**^	0.18^**^	(0.89)	
(8) Intention to Purchase	3.06	0.96	0.22^**^	−0.19^**^	0.24^**^	−0.18^**^	0.39^**^	0.34^**^	0.32^**^	(0.89)

N = 320;

** p < 0.01;

* *p < 0.05 (2-tailed), SD, Standard Deviation*.

#### Common method issues

Given that infotainment, irritation, promotion focus, prevention focus, subjective norms, perceived behavioral control, attitudes, and intention to purchase were obtained from the same sources, a potential common method variance was a concern. Several techniques were used to minimize any common source bias. First, the confidentiality of the responses was guaranteed to the participants to limit any concerns, such as the participants' evaluation apprehension or social desirability. Second, a psychological separation was constructed in the survey by using different instructions and positioning the variables in various parts of the survey with a number of filler items between them to try to minimize the respondents' perception of any direct connection between the variables (Podsakoff et al., 2003). Finally, the variance explained by the Harmon one-factor test was 19.63%, which was below the cut-off value of 25%. Hence, common method variance was an unlikely constraint of the results in this study (Peng et al., 2016).

### Test of hypotheses

#### Hypothesized model

We used the structural equation modeling technique (Anderson and Gerbing, 1988) to examine the hypotheses of this study. This technique allows a simultaneous examination of an entire system of variables in a hypothesized model (Byrne, 2009). As presented in Table 4, $\chi_{\left(356\right)}^{2}$ = 512.33, *p* < 0.001, CFI = 0.97, TLI = 0.96, and RMSEA = 0.04, the empirical results indicated that the hypothesized model fit the data well.

**Table 4:** Comparisons of the structural equation models.

**Model and structure**	**χ^2^**	***df***	**TLI**	**CFI**	**RMSEA**	**Δχ^2^ (Δ*df*)^a^**
***Hypothesized model***	512.33	356	0.96	0.97	0.04	–
***Nested model 1*** (Adding a path from Infotainment to Intention to Purchase)	512.32	355	0.96	0.97	0.04	0.01 (1)
***Nested model 2*** (Adding a path from Infotainment^*^Promotion Focus to Intention to Purchase)	510.84	355	0.96	0.97	0.04	1.49 (1)
***Nested model 3*** (Adding a path from Promotion Focus to Intention to Purchase)	511.17	355	0.96	0.97	0.04	1.15 (1)
***Nested model 4*** (Adding a path from Irritation to Intention to Purchase)	510.05	355	0.96	0.97	0.04	2.28 (1)
***Nested model 5*** (Adding a path from Irritation^*^Prevention Focus to Intention to Purchase)	511.00	355	0.96	0.97	0.04	1.33 (1)
***Nested model 6*** (Adding a path from Prevention Focus to Intention to Purchase)	512.26	355	0.96	0.97	0.04	0.07 (1)

N = 320; ^**^p < 0.01; ^*^p < 0.05 (2-tailed);

a *Compared to the hypothesized model*.

#### Model comparisons

The change in chi-square test (Bentler and Bonett, 1980) was utilized to compare the hypothesized model with nested models, which were less likely but nevertheless plausible based on theoretical arguments (Wang and Chen, 2005). Nested Models 1–6 were included.

Nested Model 1 assessed the same path as in the hypothesized model, along with the direct path from infotainment to intention to purchase. Nested Model 2 also tested the direct path from infotainment^*^promotion focus to intention to purchase in addition to the hypothesized paths. Nested Model 3 tested the direct path from promotion focus to intention to purchase in addition to the hypothesized paths. Nested Model 4 examined the direct path from irritation to intention to purchase based on the hypothesized model. Nested Model 5 examined the direct path from irritation^*^prevention focus to intention to purchase in addition to the paths of the hypothesized model. Nested Model 6 also checked the direct path from prevention focus to intention to purchase along with the paths of the hypothesized model.

The change in chi-square tests indicated that Nested Model 1 (Δχ^2^ = 0.01; Δdf = 1; *p* > 0.05), Nested Model 2 (Δχ^2^ = 1.49; Δdf = 1; *p* > 0.05), Nested Model 3 (Δχ^2^ = 1.15; Δdf = 1; *p* > 0.05), Nested Model 4 (Δχ^2^ = 2.28; Δdf = 1; *p* > 0.05), Nested Model 5 (Δχ^2^ = 1.33; Δdf = 1; *p* > 0.05), and Nested Model 6 (Δχ^2^ = 0.07; Δdf = 1; *p* > 0.05) were not significantly better than the hypothesized model and were less parsimonious (see Table 4). Hence, the hypothesized model was the most parsimonious.

Table 5 shows the results of the hypothesized model. The path coefficients were all significant. Infotainment (β = 0.21, *p* < 0.01) was positively related to attitudes, whereas irritation (β = −0.18, *p* < 0.01) was negatively related to attitudes. Thus, Hypotheses 1 and 2 were both supported. In addition, intention to purchase was related to attitudes (β = 0.27, *p* < 0.01), perceived behavioral control (β = 0.24, *p* < 0.01), and subjective norms (β = 0.27, *p* < 0.01). Hence, Hypotheses 3, 4, and 5 were supported. In addition, subjective norms were positively connected with attitudes (β = 0.19, *p* < 0.01). Thus, Hypothesis 6 was supported.

**Table 5:** Path coefficients.

**Path**	**Weight**	**S.E**.
Infotainment→AT	0.21^**^	0.05
Irritation→AT	−0.18^**^	0.04
Promotion Focus→AT	0.26^**^	0.10
Prevention Focus→AT	−0.20^**^	0.04
INTER1→AT	0.23^**^	0.05
INTER2→AT	−0.23^**^	0.04
AT→ITP	0.27^**^	0.05
PCB→ITP	0.24^**^	0.06
SN→ITP	0.27^**^	0.08
SN→AT	0.19^**^	0.07

*N = 320*,

** *p < 0.01 (2-tailed)*,

AT = attitudes toward mobile text advertising, INTER1 = infotainment ^*^ promotion focus, INTER2 = irritation ^*^ prevention focus, SN = subjective norms, PBC = perceived behavioral control, ITP = intention to purchase.

#### Moderation effect

As shown in Table 5, promotion focus (β = 0.26, *p* < 0.01) was positively related to attitudes, and the interaction between infotainment and promotion focus (β = 0.23, *p* < 0.01) was also positively related to attitudes. Thus, H7a was supported. Prevention focus (β = −0.20, *p* < 0.01) was negatively related to attitudes, as was the interaction between irritation and prevention focus (β = −0.23, *p* < 0.01). Hence, H7b was supported.

**Figure 3:**
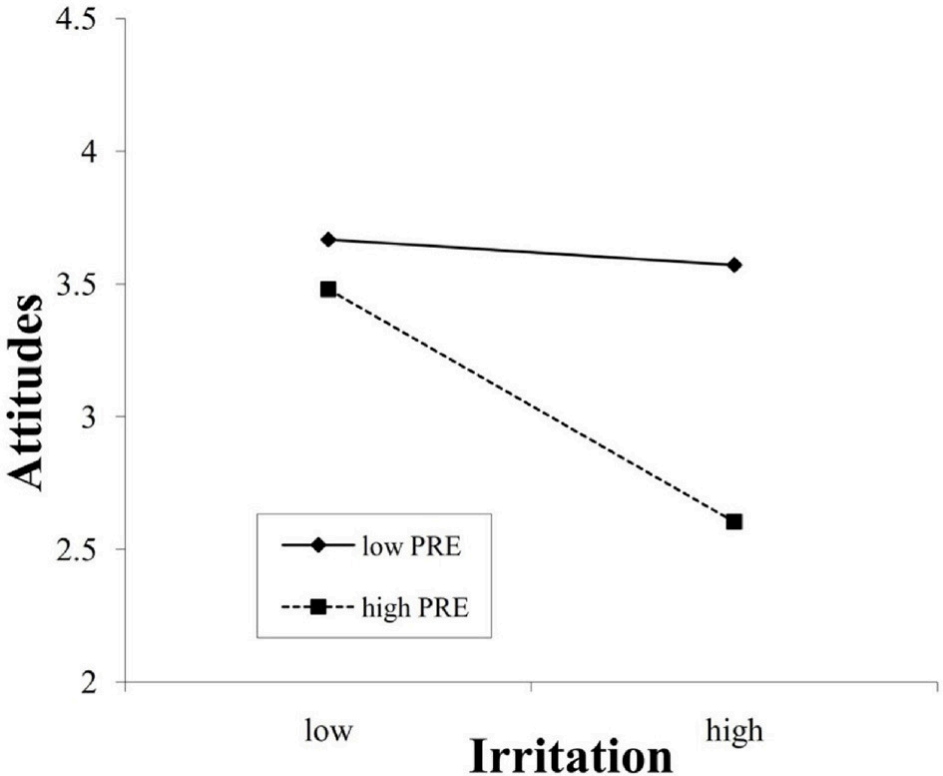
Interaction between irritation and a prevention focus in predicting attitudes.

Furthermore, the interaction effects were plotted using procedures from Aiken et al. (1994) and Dawson (2014). As shown in Figure 2, infotainment was positively related to attitude when promotion focus was high (β = 0.49, *p* < 0.01), and this positive influence was lessened when promotion focus was low (β = 0.14, *p* < 0.05), further supporting Hypothesis 7a. Moreover, Figure 3 indicates that irritation was negatively related to attitude (β = −0.44, *p* < 0.01) when prevention focus was high but was unrelated to attitude (β = −0.05, n.s.) when prevention focus was low (n.s. = non-significant). Hence, Hypothesis 7b was further supported.

#### Moderated mediation effects

A moderated path approach was utilized to test the moderated mediation hypotheses (Edwards and Lambert, 2007), and 1,000 samples were bootstrapped to compute the bias-corrected confidence intervals. An indirect effect can be viewed as statistically significant if the 95% confidence interval excludes zero (Edwards and Lambert, 2007). As shown in Table 6, an indirect effect of infotainment on intention to purchase via attitude was positive and significant when promotion focus was high (*path coefficient* (*P)* = 0.20, 99.5% CI [0.07, 0.39]) and was also significant when promotion focus was low (*P* = 0.05, 95% CI [0.01, 0.12]; difference = 0.15, 99.5% CI [0.02, 0.33]). Similarly, Table 7 shows that the indirect effect of irritation on intention to purchase via attitude was negative and significant when prevention focus was high (*P* = −0.11, 99.5% CI [−0.22, −0.01]) but was non-significant when prevention focus was low (*P* = −0.01, 95% CI [−0.06, 0.02]; difference = −0.10, 95% CI [−0.17, −0.03]). These results provide support for Hypotheses 8a and 8b.

**Table 6:** The results of moderated path analysis for Infotainment.

**Moderator variable**	**Infotainment (X)→Attitudes (M)→Intention to Purchase (Y)**				
	**Stage**		**Effect**		
	**First**	**Second**	**Direct effects**	**Indirect Effects**	**Total effects**
	***P_*MX*_***	***P_*YM*_***	***(P_*YX*)_***	***(P_*YM*_ P_*MX*_)***	***(P_*YX*_ + P_*YM*_P_*MX*_)***
Simple paths for low promotion focus	0.17^*^	0.29^**^	−0.04	0.05^*^	0.01
Simple paths for high promotion focus	0.57^**^	0.35^**^	0.20^*^	0.20^**^	0.40^**^
Differences	0.40^*^	0.06	0.24^*^	0.15^**^	0.39^**^

*N = 320*;

** *p < 0.01*;

* *p < 0.05 (2-tailed)*.

*P_MX:_ path from infotainment to attitudes; P_YM_: path from attitudes to intention to purchase; P_YX_: path from infotainment to intention to purchase. Low promotion focus refers to one standard deviation below the mean of promotion focus; high promotion focus refers to one standard deviation above the mean of promotion focus. Tests of differences between the indirect and total effects were based on bias-corrected confidence intervals derived from bootstrap estimates*.

**Table 7:** The results of moderated path analysis for Irritation.

**Moderator variable**	**Irritation (X)→Attitudes (M)→Intention to Purchase (Y)**				
	**Stage**		**Effect**		
	**First**	**Second**	**Direct effects**	**Indirect Effects**	**Total effects**
	***P_*MX*_***	***P_*YM*_***	***(P_*YX*)_***	***(P_*YM*_ P_*MX*_)***	***(P_*YX*_ + P_*YM*_ P_*MX*_)***
Simple paths for low prevention focus	−0.03	0.33^**^	0.00	−0.01	−0.01
Simple paths for high prevention focus	−0.37^**^	0.31^**^	−0.16^**^	−0.11^**^	−0.28^**^
Differences	−0.33^**^	−0.020	−0.170	−0.10^*^	−0.27^*^

*N = 320*;

** *p < 0.01*;

* *p < 0.05 (2-tailed)*.

*P_MX:_path from irritation to attitudes; P_YM_: path from attitudes to intention to purchase; P_YX_: Path from irritation to intention to purchase. Low prevention focus refers to one standard deviation below the mean of prevention focus; high prevention focus refers to one standard deviation above the mean of prevention focus. Tests of differences for the indirect and total effect were based on bias-corrected confidence intervals derived from bootstrap estimates*.

## General discussion and implications

The goal of this study was to analyze empirically the influence of mobile text advertising on consumers' purchase intention. With the rapid development of smart mobile and internet technologies, mobile shopping apps have become convenient and efficient. Mobile marketing is now essential to many companies' total marketing strategies. The question arises whether mobile text advertising, sent by mobile shopping apps, facilitates recipients' purchase intention and how this process works. The findings of this study address these questions and provide insights from both theoretical and practical perspectives.

### Conclusions

First, infotainment and irritation influenced attitudes toward mobile text advertising in opposing directions in accordance with prior research (H1, H2). The present study shows that two main dimensions of U&G, infotainment and irritation, impacted attitudes in opposing directions. The impact of infotainment was greater than that of irritation. This finding confirms previous views about mobile advertising (Tsang et al., 2004). Mobile advertisements tend to have more infotainment and less irritation to increase consumer contentment (McCoy et al., 2008).

Second, the study reveals the moderation effects of regulatory focus between information content characteristics and attitudes toward mobile text advertising (H7a, H7b). In accordance with RFT, this study showed that at a high level of promotion focus, the positive relationship between infotainment and attitudes was strengthened; conversely, at a high level of prevention focus, the negative relationship between irritation and attitudes was strengthened. These results can deepen the understanding of attitudes and benefit subsequent research.

Finally, the mechanism behind the intention to purchase mobile-advertised products was explored. After receiving mobile text advertisements from mobile shopping apps, the consumers' attitudes toward mobile text advertising, together with their subjective norms and perceived behavioral controls, influenced the intention to purchase (H3, H4, and H5). Moreover, this study suggested that subjective norms indirectly impacted the intention to purchase through their attitudes toward mobile text advertising (H6). According to reference group theory, the views of others considerably affect an individual's beliefs and their purchase decisions. Our study indicated that this was true in a mobile advertising context. Additionally, two moderated mediation effects were identified (H8a, H8b). One mediation effect of attitudes toward mobile text advertising between infotainment and intention to purchase was moderated by the promotion focus; whereas, the other mediation effect of attitudes toward mobile text advertising between irritation and intention to purchase was moderated by the prevention focus.

In addition, due to the similarities between the Taobao app and other mobile shopping apps, the above conclusions are also applicable to similar apps. The Taobao app has the largest number of Chinese shoppers (QuestMobile, 2017) and also has a range of products and services which covers most online items. Estimates are that 82% of Chinese internet advertising will be invested in mobile trading platforms by the year 2021 (eMarketer, 2017). Therefore, delivering text advertising via apps can be expected to become a prosperous method. Our findings about mobile text advertising via the Taobao app can be expected to be basically stable.

### Theoretical implications

First, our findings shed light on the use of (promotion focus and prevention focus) regulatory focus in mobile advertising activities. Previous studies on mobile advertising were mostly based on either the single theory of reasoned action (Tsang et al., 2004) or the theory of acceptance model (Muk and Chung, 2015). In the present study, the regulatory focus was found to not only significantly influence the relationship between information characteristics and attitudes but also to moderate the mediation effects of attitudes on information characteristics and the intention to purchase. The empirical test showed that a high promotion focus strengthened the impact of infotainment on attitudes as well as that attitudes mediated the relationship between infotainment and the intention to purchase. Moreover, a high prevention focus increased the impact of irritation on attitudes in addition to reinforcing the mediated effect of attitudes between irritation and the intention to purchase. The results can not only contribute to the further understanding of consumer behavior characteristics with regard to the mobile field but can also shed light on related research based on the RFT in the context of mobile commerce. Furthermore, this study extends the application of RFT to the mobile context and other mobile service activities.

Second, this study helps to elucidate the mechanism of the purchase intention in the mobile context, indicating that it is an extension of the TPB. Most extant studies have discussed the intention to purchase only from the perspective of the TPB. This study, which investigated the intention to purchase from the perspective of regulatory focus, identified a moderating role for regulatory focus, mediating effects of attitudes, and moderated mediation effects. The results of this study improve the understanding of the mechanism underlying the intention to purchase in a mobile context. The results also highlighted the importance of consumer motivation in the mobile field. Moreover, this study laid a foundation for conducting related behavioral intention research in different contexts.

Third, the results of our research appear to be representative and universal. Extant studies on mobile advertising have primarily focused on no-income groups, such as university students. In this study, 95% of the respondents had had mobile shopping experience for at least 1 year and 76% were employees from various companies and thus had a certain economic foundation. Hence, we believe that our data is representative and our findings will be valuable. Furthermore, the empirical results showed that the participants had either a positive attitude (Table 4, the attitudes mean = 3.32) or a near neutral irritation (Table 4, irritation mean = 2.94), a finding which is consistent with the notion that the negative impact of irritation has become weaker than in the past (Xu et al., 2009).

### Practical implications

In addition to the above theoretical implications, this study has the following practical implications.

First, the findings highlighted the importance of subjective norms. Although attitudes are usually emphasized in consumer behavior intention research, managers must carefully attempt to govern the influence of reference groups on attitudes and the intention to purchase in the mobile context.

Second, the findings can enlighten designers about the design of mobile advertising. Managers often try to avoid irritating consumers. Our results suggest that they should also concentrate on increasing the infotainment to please consumers. Avoidance alone is not enough. A high level of infotainment is beneficial to the formation of a positive attitude and to the purchase intention. Advertising designers may need to increase the informativeness and entertainment of mobile advertising content.

Third, the findings can contribute to perfecting the marketing strategy of various companies. In general, marketing managers employ strategies aimed at managing the information content characteristics of mobile advertising (Tsang et al., 2004; Xu et al., 2009; Liu et al., 2012). This study suggested that they should pay attention not only to the information content of mobile advertising but also to the regulatory focus (promotion focus and prevention focus). For example, delivering enjoyable mobile text advertising to promotion focus customers may contribute to creating better attitudes and a stronger intention to purchase; but how to identify promotion focus customers and prevention focus ones is the question. The literature indicates that a promotion focus can be triggered temporarily in some situations (Lockwood et al., 2002). Managers could motivate consumers to purchase by activating each individual's promotion focus by some mobile promotion activities. Furthermore, the method we used in this article can serve as a reference for managers. By sending the online testing items of about a regulation focus to their customers, they may be able to distinguish between them. Analyzing the existing customer database is another possible method (Das, 2016). In addition, promotion focus individuals are inclined to change the current situation, whereas prevention focus ones are inclined to maintain the status quo (Chernev, 2006; Herzenstein et al., 2007). Hence, managers could deliver the customized information about their goods in accordance with the recipient's regulatory focus. For instance, for the promotion focus customers, receiving new or high technology products text advertising from shopping apps would help to draw attention and improve the products' brand awareness, whereas sending information about how the product will help the recipients maintain their status quo will tend to attract prevention focus individuals.

## Limitations and future research

Although, all of the hypotheses in this study were supported, the study still presents limitations that can provide opportunities for future research. First, only push mobile advertising was examined. Future research should explore the effectiveness of pull mobile advertising and compare the two forms. Second, the study only concentrated on the text form of mobile advertising. In the future, other types of mobile advertising, such as multimedia mobile advertising, should be investigated as well. Third, this study explored the moderation effect only in a mobile environment. The RFT's influence in the content-attitudes relationship, as well as its moderation effect in the attitudes-intentions relationship, is worth studying as a general TPB modifier as well as in a mobile advertising context in further scenarios. In addition, our analysis may lag behind the rapid development of mobile advertising and communication technology to some extent. Because of these rapid advances, the formation mechanism of the intention to purchase will need to be continually updated. Fourth, we primarily included subjects who were fairly well-educated and investigated their responses to a single shopping app. According to statistics from the China Internet Network Information Center (CNNIC, 2017), college students are the major users of Chinese cybercitizens. However, a further study utilizing a different range of the population and more shopping apps should also be performed to strengthen its robustness. Finally, the study discussed chronic promotion and prevention focuses, but these may have been triggered in some situations. Future research should examine the manner in which they can be triggered and the things that influence them.

## Ethics statement

This research was approved by Economics and Management Department Research Ethic Committee. All participants gave informed consent to participate. All participants were fully insured for the duration of the questionnaire.

## Author contributions

LH contributes the writing of the whole article. CZ contributes the data collection.

### Conflict of interest statement

The authors declare that the research was conducted in the absence of any commercial or financial relationships that could be construed as a potential conflict of interest.

## Abbreviations

Apps: applications
SMS: short message service
U&G: uses and gratifications
TPB: theory of planned behavior
RFT: regulatory focus theory.
